# COVID-19 vaccine race: watch your step for cancer patients

**DOI:** 10.1038/s41416-020-01219-3

**Published:** 2020-12-07

**Authors:** Raphaelle Fanciullino, Joseph Ciccolini, Gerard Milano

**Affiliations:** 1 Pharmacy Unit, La Conception, University Hospital of Marseille, APHM, Marseille, France; 2 SMARTc Unit, Centre de Recherche en Cancérologie de Marseille Inserm U1068 Aix Marseille Université and Assistance Publique Hôpitaux de Marseille, Marseille, France; 3 Oncopharmacology Unit and UNS EA 7497 Nice University, Centre Antoine Lacassagne, Nice, France

**Keywords:** Health policy, Cancer immunotherapy

## Abstract

Patients with cancer should benefit from COVID-19 vaccination. Some of the most advanced vaccine candidates are mRNAs encapsulated into lipid carriers, and small liposomes are expected to accumulate in tumour tissues through the enhanced and permeation retention effect. However, to what extent solid tumours could take up a significant part of the vaccine dose as well remains unknown. This calls for a careful evaluation of the efficacy of these promising mRNA COVID-19 vaccines administered as lipid carriers for patients with solid tumours, including a possible re-appraisal of the dosing for optimal protection of this specific and frail population.

## Main

The prolonged severe acute respiratory syndrome coronavirus 2 (SARS-CoV-2) (coronavirus disease 2019 (COVID-19)) pandemic has prompted the worldwide development of vaccine candidates at an accelerated pace, and >180 projects have currently reached clinical evaluation phases.^1^

The identification of SARS-CoV-2 Spike (S) Protein targeting cellular angiotensin-converting enzyme 2^2^ has led to the development of two categories of vaccines targeting this protein. The first one is based on an inactivated virus expressing the S Protein following a specific genetic manipulation. The second one relies on the injection of nucleotides (i.e., DNA or mRNA) guiding the synthesis of the S Protein by the organism.

Thus far, the mRNA forms were the first to reach Phase 3 trials with promising efficacy. For physico-chemical and biological stability reasons, S Protein mRNA must be encapsulated into liposomes and delivered as lipid nanoparticles. Among the priority populations to benefit from COVID-19 vaccination will be the most fragile individuals, such as cancer patients, for whom COVID-19 can be particularly detrimental.^3^

There is a decade-old history of entrapping anticancer agents into liposomes as drug carriers in patients with cancer, including delivering nucleotides such as small interfering RNA.^4^ Liposomes are expected to accumulate in tumour tissues by passive targeting, a phenomenon best known as the enhanced permeation and retention (EPR) effect. Most tumour vasculatures are indeed characterised by 200 nm fenestrations because of non-junctive endothelial cells, a reduced layer of smooth muscle, a lack of homoeostatic blood flow control, reduced expression of angiotensin II receptors and high levels of vascular permeability factors.^5^ All of these characteristics explain how leaky neo-vessels can be in the tumour surroundings, thus making nanoparticles easily leave the blood flow and reach cancer cells provided that their size is <200 nm. Reduced lymphatic drainage further explains why nanoparticles will accumulate in the tumour micro-environment after leaking out of the blood vessels.^6^ Experimental data in tumour-bearing animals have already shown how the EPR effect was dependent on liposome size, i.e. the smaller the size, the higher the tumour accumulation. Usually, the EPR effect leads to 5–10% of the injected dose to be found in tumours for larger nanoparticles, and up to 25% for the smaller ones, at least in animal models.^7^ Interestingly, a positive correlation was found as well between liposome accumulation in tumours and vascular density: the denser the vessels, the greater the tumour uptake.^7^ In humans, the EPR effect has been confirmed, i.e. higher drug concentrations in solid tumours were observed in patients with cancer treated with liposomal cytotoxic agents as compared with those with free drug administration.^8^ With respect to the fact that some of the most advanced COVID19 vaccine candidates are based on mRNA delivery using liposomes, this raises the question of possible uptake of those vaccines by tumour tissues as well. Indeed, as for most proteins delivered using lipid carriers, two of the upfront vaccine candidates (i.e. the Moderna mRNA-1273 and the Pfizer BNT162b2) are both delivered as small (i.e. <200 nm) nanoparticles.^9,10^ Therefore, they are theoretically prone to the EPR effect as well, with a possible significant accumulation in tumour surroundings, as for any other drug given as liposomes. To what extent this specific delivery towards cancer cells could be an issue in patients with solid tumours is unknown and therefore remains to be fully evaluated. Indeed, part of the vaccine dose could be captured by tumours, rather than being properly and rapidly distributed to the spleen to trigger an immunogenic effect. This could lead to a possible modification of protection against COVID-19, and the results of early-phase clinical trials have already suggested the importance of dosing for effective COVID-19 protection.^10^ Consequently, to what extent COVID-19 vaccine dosing should be re-appraised with respect to a possible uptake of the liposomes by tumour tissues merits consideration. COVID-19 vaccines delivered as lipid carriers are administered by the intra-muscular (IM) route, and preclinical data have already shown that proteins encapsulated into liposomes and given via IM administration lead to systemic distribution in the body.^11^ Another possible consequence of the specific delivery of the S Protein as a liposomal form to cancer cells could be a modification of tumour immunity, with subsequent potential impact on disease evolution and a possible change in sensitivity to immunotherapy. Because these effects remain unknown as well, this calls for specific studies deciphering the impact of liposomal vaccines on tumour biology. Careful evaluation of the efficacy of these promising mRNA COVID-19 vaccines administered as lipid carriers should thus apply for patients with solid tumours, including a possible re-appraisal of the dosing for optimal protection of this specific population. In the meantime, should a large-scale vaccination campaign be started urgently in patients with cancer, considering alternative forms of COVID-19 vaccines (i.e. not administered as liposomes) would be reasonable at least for patients with solid tumours. The panel of alternative vaccines reaching the final development phases is extending and this would allow cancer patients to benefit from a large-scale vaccination campaign against COVID-19, at least until comprehensive knowledge regarding the specific distribution of vaccines administered as liposomes in patients with solid tumours is gained.

## Data Availability

Not applicable.

## References

[1] Krammer F (2020). SARS-CoV-2 vaccines in development. Nature.

[2] Brest P, Refae S, Mograbi B, Hofman P, Milano G (2020). Host polymorphisms may impact SARS-CoV-2 infectivity. Trends Genet..

[3] Gosain R, Abdou Y, Singh A, Rana N, Puzanov I, Ernstoff MS (2020). COVID-19 and cancer: a comprehensive review. Curr. Oncol. Rep..

[4] Boca S, Gulei D, Zimta AA, Onaciu A, Magdo L, Tigu AB (2020). Nanoscale delivery systems for microRNAs in cancer therapy. Cell. Mol. Life Sci..

[5] Van Eerden RAG, Mathijssen RHJ, Koolen SLW (2020). Recent clinical developments of nanomediated drug delivery systems of taxanes for the treatment of cancer. Int. J. Nanomed..

[6] Maeda H, Bharate GY, Daruwalla J (2009). Polymeric drugs for efficient tumor-targeted drug delivery based on EPR-effect. Eur. J. Pharm. Biopharm..

[7] Fanciullino R, Mollard S, Correard F, Giacometti S, Serdjebi C, Iliadis A (2014). Biodistribution, tumor uptake and efficacy of 5-FU-loaded liposomes: why size matters. Pharm. Res..

[8] Atrafi F, van Eerden RAG, van Hylckama Vlieg MAM, Oomen-de Hoop E, de Bruijn P, Lolkema MP (2020). Intratumoral comparison of nanoparticle entrapped docetaxel (CPC634) with conventional docetaxel in patients with solid tumors. Clin. Cancer Res..

[9] Pardi N, Tuyishime S, Muramatsu H, Kariko K, Mui BL, Tam YK (2015). Expression kinetics of nucleoside-modified mRNA delivered in lipid nanoparticles to mice by various routes. J. Control Release.

[10] Mulligan MJ, Lyke KE, Kitchin N, Absalon J, Gurtman A, Lockhart S (2020). Phase I/II study of COVID-19 RNA vaccine BNT162b1 in adults. Nature.

[11] Li H, Yang L, Cheng G, Wei HY, Zeng Q (2011). Encapsulation, pharmacokinetics and tissue distribution of interferon alpha-2b liposomes after intramuscular injection to rats. Arch. Pharm. Res..

